# Assessing seed priming, sowing date, and mulch film to improve the germination and survival of direct‐sown *Miscanthus sinensis* in the United Kingdom

**DOI:** 10.1111/gcbb.12518

**Published:** 2018-06-07

**Authors:** Chris Ashman, Danny Awty‐Carroll, Michal Mos, Paul Robson, John Clifton‐Brown

**Affiliations:** ^1^ Institute of Biological, Environmental and Rural Sciences Aberystwyth University Aberystwyth UK; ^2^ Terravesta Lincoln UK

**Keywords:** agronomy, bioenergy, direct seed sowing, *Miscanthus*, modelling, seed biology, seed germination

## Abstract

Direct sowing of *Miscanthus* seed could lower crop establishment costs, and increase the rate of grower uptake and biomass supply for the emerging bio‐economy. A replicated field trial was conducted at two contrasting UK sites: Aberystwyth (ABR) in mid‐Wales and Blankney (BLK) in Lincolnshire. These sites encompass the west–east meteorological gradient in the United Kingdom where the growing season at ABR is cooler and wetter while BLK is warmer and drier. Primed and unprimed *Miscanthus sinensis* seeds were sown directly onto the soil surface with and without a clear biodegradable mulch film, at nine dates interspersed from May to October. Average daily mean soil surface temperatures measured over the first 2 months after sowing under the mulch film were higher than control plots (2.7°C ABR and 4.2°C BLK). At both sites, the film covering also affected soil volumetric moisture relative to uncovered control plots (−3% ABR and 8% BLK), demonstrating the negative impact of mulch film when sowing on dry soil. Over nine sowings, seed germination at ABR under film varied between −28% and +18% of germination under control conditions. Seedlings from the first three sowings at both sites under film had sufficient physiological maturity to survive the first winter period. At BLK, mulch film significantly increased tiller count and height in both the first and second years after sowing. At ABR, where temperatures were lower, film covering significantly increased tiller height but not count. Water priming had no significant effect on seed viability or germination in the field tests. Base temperatures for germination of primed and unprimed seeds on a thermal gradient plate were 7.0°C and 5.7°C, respectively, with a ± 1.7°C confidence interval. Based on our results for *M. sinensis* in the United Kingdom, we recommend the sowing of unprimed seed in May under film and only when the soil is moist.

## 1 INTRODUCTION

*Miscanthus* is a genus of rhizomatous, perennial *C*_4_ grass originating from Asia (Hodkinson, Chase, Lledó, Salamin, & Renvoize, 2002; Stewart et al., 2009). It is a second‐generation energy crop with strong prospects for use as a biofuel (Christian, Yates, & Riche, 2009). *Miscanthus* produces high yields with minimal inputs and can grow on marginal land unsuitable for other arable agricultural practices, thus avoiding the potential conflict with land required for food production (Valentine et al., 2012).

Currently, the most common commercially grown *Miscanthus* is a sterile triploid that can only be propagated clonally: *Miscanthus* × *giganteus Greef et Deu* (Aksel Olson) (Hodkinson & Renvoize, 2001) (*M *× *g*). The most common commercial propagation method is by rhizome (McCalmont et al., 2015). Rhizome production needs large nursery fields (approximately 1 ha generates sufficient rhizome to plant 13 ha in the United Kingdom), and each nursery plot takes around 2 years to recover productivity after partial rhizome removal from the mother field. This adds to the cost and reduces the speed with which *Miscanthus* is deployed across large areas. More rapid propagation methods for *M *× *g* include in vitro tillering (Lewandowski, 1997), but the costs for widespread upscaling are prohibitive at ~6,000 euro/ha (Xue, Kalinina, & Lewandowski, 2015). Seed propagation is much more scalable than clonal propagation because multiplication rates up to 2,000 have been reported from experimental seed production trials with novel interspecific hybrids (Clifton‐Brown et al., 2016). However, to establish *Miscanthus* from seed is not trivial in temperate climates because the seeds are small and have a high thermal requirement for germination (Hsu, 1985), and initially grow slowly compared to *C*_3_ weeds. To overcome these barriers, the commercial upscaling experiments to date have focused on the use of glasshouse‐grown plug plants, which are transplanted in early spring into the field. Projections of the likely cost savings, relative to rhizome planting *M *× *g*, using “plug” planting show reductions of about 20%–40% in planting costs are realistic (Hastings et al., 2017).

To reduce the costs further, it will be necessary to develop methods for direct sowing of seed to the soil. In this study, we explore several agronomic techniques applicable to direct drilled *Miscanthus* seed. To establish *Miscanthus* from direct‐sown seed, the thermal and moisture requirements to initiate germination must be met (Clifton‐Brown et al., 2011) and the resulting plants must, across the remaining season after germination, experience conditions necessary to produce sufficient growth and phenological development to survive the first winter. Sowing time will affect the moisture and temperature the seed and seedling experience. The thermal requirements for germination and accumulation of biomass through a long growth season may act in opposition (Hastings et al., 2017). This may be particularly so in temperate regions, where the differential in temperature throughout the year is high and earlier sowings are subject to low temperatures. Christian, Yates, and Riche (2005) tested the direct drilling and broadcasting of natural and coated *Miscanthus* seed in field trials. They found that although *Miscanthus sinensis* seed emerged within 10 days of sowing, high seedling death occurred from seedling desiccation if conditions were dry three to 4 weeks after sowing. Direct drilling was shown to be more successful than broadcasting, and uncoated seed had higher germination rates than pelleted seed.

Clifton‐Brown et al. (2011) compared the base temperature needed for 50% germination of viable seed in 10 *M. sinensis* families, switchgrass (*Panicum virgatum*), reed canary grass (*Phalaris arundinacea*), perennial ryegrass (*Lolium perenne*), and maize (*Zea mays*). They found that perennial ryegrass and maize had the lowest base temperatures at 3.4 and 4.5°C, respectively. Base temperatures within the *Miscanthus* families ranged from 9.7°C to 11.6°C. They predicted that seed establishment of *Miscanthus* in Northern Europe during spring was unlikely to be viable unless soil temperatures could be raised artificially.

Crops such as winter barley (*Hordeum vulgare*) or winter wheat (*Triticum aestivum*) can be sown from mid‐September (Pillinger, Evans, Whaley, Knight, & Poole, 2004; Rasmussen, 2004). Sowing at this time reduces workload during intensive months of spring and would allow seed to be sown into a warm seed bed. However, there are risks, soil surface moisture may be too low, and immature seedlings may not survive through winter. For these reasons, a spring to early summer sowing is likely to me more successful. To maximise the growing season and improve germination and establishment conditions, agronomic techniques available for other spring‐sown crops may be adapted for *Miscanthus*.

Forage maize is a *C*_4_ crop that needs a long growing season to fully mature. In semiarid or cooler climates that are less favourable for growing maize, mulch films, made from a clear or coloured starch that is degraded by light or microbes, have been used to increase soil temperature and retain soil moisture (van der Werf, 1993) and significantly increase maize yields in either semiarid (Zhou, Li, Jin, & Song, 2009) or cooler climates (Farrell & Gilliland, 2011). The increase in yield is achieved by reducing evaporation from the soil and increasing the soil temperature (Easson & Fearnehough, 2000). The mulch film creates a “greenhouse effect” by which incoming solar radiation passes through the film, trapping warmed air between the soil and the film and warming the soil surface (Zhou et al., 2009). Trials with *M *× *g* in Ireland have shown benefits of film establishment are still detectable 3 years later (O'Loughlin, Finnan, & McDonnell, 2017).

Seed priming is widely used to improve germination (Sharma, Rathore, Srinivasan, & Tyagi, 2014) and is increasingly common in industrial agriculture (Sathish, Sundareswaran, & Ganesan, 2011). Priming initiates seed germination but suspends germination in phase two (lag phase), improving seedling vigour in some species (Hussian et al., 2014). Water priming has been shown to reduce the thermal time to germination and improve the consistency of germination in onion (*Allium cepa*) (Ellis & Butcher, 1988). However, water priming can age seed faster (Hacisalihoglu, Taylor, Paine, Hilderbrand, & Khan, 1999), and despite an improvement in establishment uniformity and vigour in some cases, there is a risk of reducing vigour (Sathish et al., 2011).

If directly sowing seed resulted in slower plant development than alternative establishment methods, resulting in a longer establishment period to reach economically harvestable yield levels, direct sowing would not be commercially viable. This study aimed to assess the effect of seed priming, mulch film, and sowing date on the germination and establishment of *M. sinensis*. We hypothesise that mulch film will stimulate germination especially at early sowings and will aid establishment by reducing soil moisture losses. We expect priming to reduce the base temperature (*T*_b_) and increase germination at lower temperatures. Evaluating these factors will help to determine whether the use of common agronomic techniques could provide the “know‐how” to establish seed‐based *Miscanthus* hybrids by direct sowing.

## 2 MATERIALS AND METHODS

### 2.1 Details of field sites and climates

The trials were conducted at two locations: Aberystwyth (ABR) (52°24′53.8″ N, 004°02′31.7″ W) and Blankney (BLK) (53°06′59.5″ N, 000°23′35.4″ W). Seed establishment is known to be particularly climate dependent, therefore, testing at multiple sites is common (Parrish & Fike, 2005) and increases the range of climatic variables, and highlights the effect of soil conditions on seed establishment.

The soil surface texture in ABR is sandy loam while in BLK it is clay loam. The 10‐year averages for minimum and maximum temperatures were 6.6°C and 13.8°C, respectively, for BLK and 7.6°C and 12.7°C, respectively, for ABR. Ten yearly average rainfall was 622 mm for BLK and 924 mm for ABR (Data from Met Office sites Waddington 14 km from BLK and Aberporth 47.8 km from ABR).

### 2.2 Experimental design and agronomic treatments

A randomised block design with three replicates was developed using a random number generator and sown at intervals of 3 weeks, across nine sowing dates between May and September 2013 with split plots for four treatments: primed seed, unprimed seed, mulch film and no mulch film.

*Miscanthus sinensis* seed from a synthetic population was produced and machine threshed in the United States by CERES Inc. (Thousand Oaks, California) in autumn 2012. The seeds were water primed by Elsoms Seeds Ltd. (Spalding, Lincolnshire, United Kingdom); in this method, the seeds were imbibed in slowly rotating drums with water at the bottom until almost chitted then dried back to their starting moisture content. The time taken for chitted seeds to be observed was determined in a previous experiment using the same seed batch. Control seed and seed for priming were taken from the same seed batch. The mulch film used was Samco “Grey” film with “pin hole 20” aeration (Samco Agricultural Manufacturing Ltd., Adare, Limerick, Ireland). The pin hole system (two rows of perforations with 75 cm between each row of perforations) improves ventilation and helps the plants to push through the film. Samco “Grey” is a 7‐micron‐thick starch film that is degraded by temperature, UV and soil microbes (Samco Ltd, 2014).

**Figure 1:**
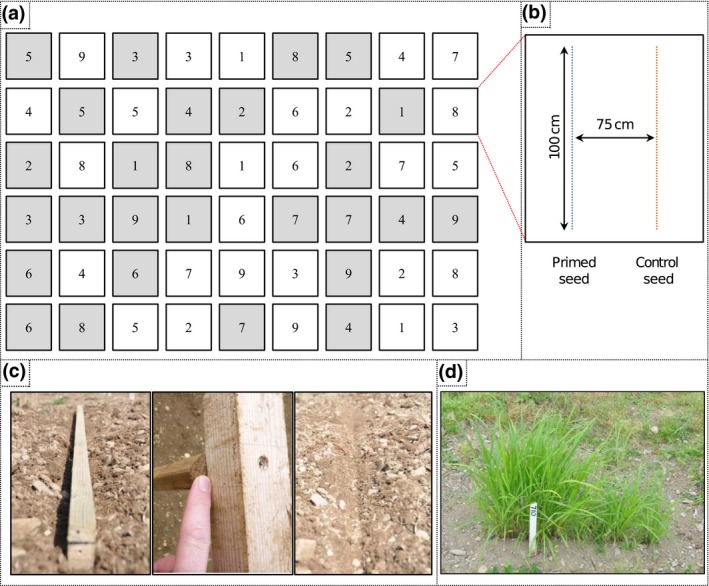
An RCBD design with split plots was used. (a) Trial design, at each sowing date six plots were sown, three were covered with mulch film (grey) while three were control (uncovered), one per block of each treatment. Genstat 14th edition was used to randomise the treatments per block. (b) Split plots were used, each plot contained one row of primed seed and one row of control seed. Each row was 100 cm long, and the inter‐row distance was 75 cm. Three hundred seeds were sown by hand in each row. (c) A wooden frame was used to produce a suitable seed bed and ensure the inter‐row distance was correct. (d) Weeds were removed by hand within the plot. Grass was allowed to grow in the paths and controlled by mowing

The sites were prepared by spraying with Roundup™ (Syngenta, Fulbourn, UK), ploughed, then power harrowed or rotavated depending on site to produce a fine tilth. An CRD design with split plots was used. Six plots were sown at each sowing, and immediately after sowing, three plots were covered with mulch film leaving three uncovered (control) plots (Figure 1a). Genstat 14th edition was used to randomise the treatments. Before each new sowing, the seedbed of the plots sown was recultivated to a fine tilth. Each plot contained two rows of seeds: one row of primed seed and one of unprimed control seed (from the same seed batch as the primed seed) (Figure 1b). Prior to sowing, a suitable seedbed was created by compressing the soil into 1‐cm‐deep “V”‐shaped 1‐m‐long furrow using a wooden frame (Figure 1c). Each furrow was 1 m long and contained 300 seeds which were evenly distributed along the furrows by hand sowing. The inter‐row distance between the rows of seed within the plots was 75 cm which aligned with the “pin holes” in the mulch film. The mulch film was laid manually and secured by digging a ditch around the edge of individual plots which were backfilled with soil to secure the edges of the film.

It is normal commercial practise to apply a soil acting pre‐emergence herbicide prior to covering with film; however, due to the lack of permitted *Miscanthus* safe herbicides (Anderson, Voigt, Bollero, & Hager, 2010; Everman et al., 2011), no herbicides were used in this study. Any remaining weeds present were removed from the plots by hand (Figure 1d).

### 2.3 Environmental and morphological measurements

Volumetric soil moisture content and soil temperature were monitored using water content reflectometers (Campbell Scientific CS616) and type T (copper–constantan) thermocouples connected to a Campbell CR1000 data logger (Campbell Scientific Ltd., Loughborough, UK). At the first sowing, reflectometers and thermocouples were positioned horizontally at sowing depth (~1 cm covering of the probes) in two film‐covered plots and two in plots without film, these were averaged and are stated ±1 *SE*. Due to the positioning of the reflectometers, they could only show the relative water content of the soil because part of their range was above the soil surface. Data were recorded at hourly intervals. Rainfall and screen air temperature at 2 m were collected at both sites from January 2013, 5 months before the first sowing, until completion of the experiment. Soil water deficit was calculated using meteorological data from each site starting on the 1st of January 2013 using the Penman–Monteith equation (Monteith, 1965).

Seedling emergence counts were made approximately every 2 weeks after sowing in each plot for up to 10 weeks in ABR. As plots from different sowings were not assessed at the same number of days from sowing, the emergence counts used are averages of several seedling emergence counts within a 4‐week period. Autumn seedling vigour was assessed by tiller counts per plot and tiller heights in November 2013 and November 2014. Tiller height was the length from the ground to the youngest ligule. Tiller diameter was measured 1 cm above ground level. The number of fully emerged leaves was assessed from 5 to 10 plants (depending on establishment rate) distributed along each plot row.

### 2.4 Germination of seed across a thermal gradient

To determine the base temperature of both the control and primed seed, a germination trial was conducted under controlled conditions using a thermal gradient plate. The standard configuration of the thermal gradient plate (Grant Instruments Ltd., Cambridge, UK) was modified by replacing the Grant controllers with a CR10 data logger controller (Campbell Scientific, Loughborough, UK) as described in Clifton‐Brown et al. (2011). A 2 × 14 grid was used for testing the primed and control seed sets at 14 temperature points. A glass thermometer and a multimeter (CA5233 Chauvin Arnoux) thermocouple were used to calibrate and cross‐check the temperatures recorded by the logger at the beginning and the end of the experiment (the temperatures varied by <1°C within each treatment row‐step). To ensure the accuracy of the thermal gradient throughout the experiment, thermocouples were placed in three out of the fourteen row‐steps corresponding to approximately 5°C (coldest), 18°C (mid) and 31°C (warmest). Mean temperatures were 4.3°C, 19.5°C and 31.6°C, respectively, at set points of 5°C, 18°C and 31°C with standard deviation of 0.3°C, 0.5°C and 0.4°C, respectively.

The thermal gradient plate was set to create a temperature gradient to approximate the range of averaged soil surface temperatures measured in the field trials in 2013 from both field sites. The field temperatures fluctuated from −5°C to 42°C; however, these extremes were not maintained for extended periods, and therefore, the 6 hourly averages of the soil surface temperature were used. The six hourly average temperatures ranged from 4.6°C to 33.3°C; these were simplified to give regular spaces of 2°C per column on the thermal gradient from 5°C to 31°C. Due to the cell numbers available one replicate per temperature was used, 60 seeds were sown per cell. Seeds were placed on moist “WYPALL L30” blue‐coloured tissue paper (Kimberly‐Clark, Reigate, Surrey, UK). During the experiment seeds on the thermal gradient were kept moist by simple capillary action of the tissue paper from irrigation gutters which were checked every 48 hr and topped up as necessary.

Germination was visually assessed every day at the same time. Any obviously dead (mouldy) seeds were removed at approximately 150‐hr intervals to prevent mould spreading to other seed. Germinated seed was counted and removed once the white radical (which contrasts well with the blue tissue paper) had visibly emerged (Bewley, 1997; Ellis, Hong, & Roberts, 1985). The experiment was stopped after no further germination was recorded for 3 days. The remaining ungerminated seeds were assessed for viability using tweezers (Borza, Westerman, & Liebman, 2007), and soft/damaged seeds were removed before the temperatures across the whole gradient table were increased to 30°C for 5 days to force germination; this produced an estimate of total viable seed out of 60 (a similar approach was taken in Clifton‐Brown et al. (2011)). At the end of the experiment, all 60 seeds per treatment row‐cell were placed into five categories: germinated, mouldy, viable, nongerminated soft seed or nongerminated firm seed.

### 2.5 Statistical analysis

#### 2.5.1 Field sowing experiment

Morphological data sets of autumn seedling vigour were analysed with R (R Core Team, 2015), using three‐way ANOVAs of height, thickness and leaf number. The three factors used in the ANOVA were sowing number, film and priming. Each was analysed with interactions first, if the interactions were not close to significance, they were removed from the model and the model was run again (there were no significant interactions). The two models were then compared, and if not significantly different, the simpler model was used, unless its residuals deviated significantly from normality. Tillering was analysed in the same way as emergence, with a generalised linear model using a Poisson or negative binomial distribution (from the mass package (Venables & Ripley, 2002)), this was dependent on the result of a Vuong's test (Zeileis, Kleiber, & Jackman, 2008). To determine the effects of each treatment, a model was produced without that treatment, an ANOVA was then used to compare the likelihood ratios between the two models (Pinheiro & Bates, 2000).

#### 2.5.2 Thermal gradient experiment

Using the range of temperatures and rates of germination on the thermal gradient plate, the base temperature for the seeds was estimated by interpolation. This estimation used a logistic distribution similar to Clifton‐Brown et al. (2011), where a logistic curve was fitted to the proportion of viable seeds germinated along the range of thermal times with a default base temperature (0°C); from this curve, the point of inflection (*T*_50_) could be extracted. (1/*T*_50_) gives the rate of germination for each cell on the thermal gradient plate; a linear regression was then used to find the intercept from all the rates (1/*T*_50_) for each seed lot. Confidence intervals were bootstrapped to this intercept using estimated standard error (using the “boot” r package Davison and Hinkley (1997)).

## 3 RESULTS

### 3.1 Environmental conditions during the field experiment

Total annual rainfall in Blankney (BLK) was 444 mm and 687 mm in 2013 and 2014, respectively; this was less than in Aberystwyth (ABR) which over the same periods was 747 mm and 829 mm (Figure S1). ABR experienced unseasonably low rainfall between January and April in 2013. The mean air temperature between May 2013 and October 2013 (growing season 1) was higher in BLK at 14.8°C than in ABR which was 13.8°C; however, the first winter (November 2013–March 2014) was slightly colder in BLK (7.4°C) than in ABR (7.9°C). Rainfall was much greater at ABR than BLK throughout the first growing season (May to October), the sum of the rainfall was 315 mm and 190 mm in ABR and BLK, respectively.

**Figure 2:**
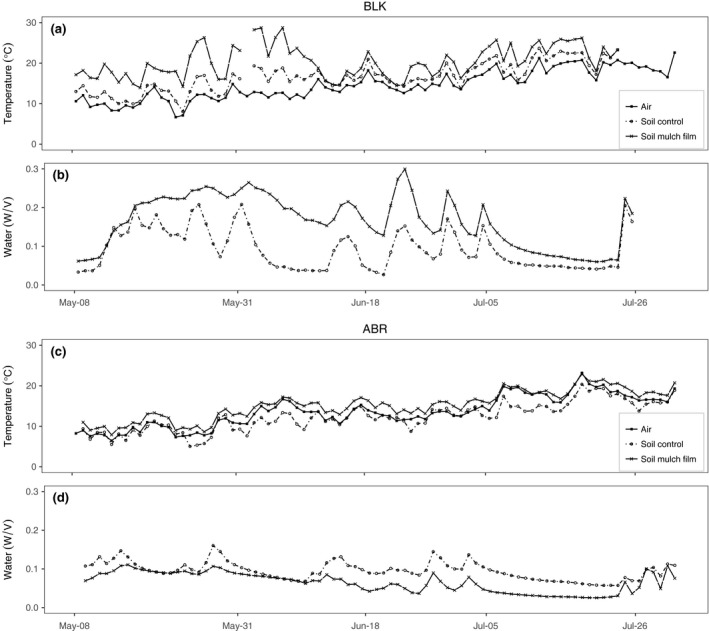
Meteorological data from the two contrasting study sites in the United Kingdom at Aberystwyth (ABR) and Blankney (BLK). Panels a and c show mean daily air temperature measured by nearby weather stations and the temperature at seed depth. Panels b and d for each site plot the soil water content at the sowing depth of the seed. The measurements of soil water and temperature were recorded from sowing time 1 plots only and are shown as an average of two sensors for control plots (no film) and two sensors for plots covered with mulch film. The film covering for these plots disintegrated before June 18th at BLK and between the 5th and the 26th of July at ABR. Therefore, after late June the comparison was ended and the trace is discontinued. Plots uncovered and covered with mulch film were set up at each sowing time, and the dates on the *x*‐axis correspond to the first 5 sowing times

**Figure 3:**
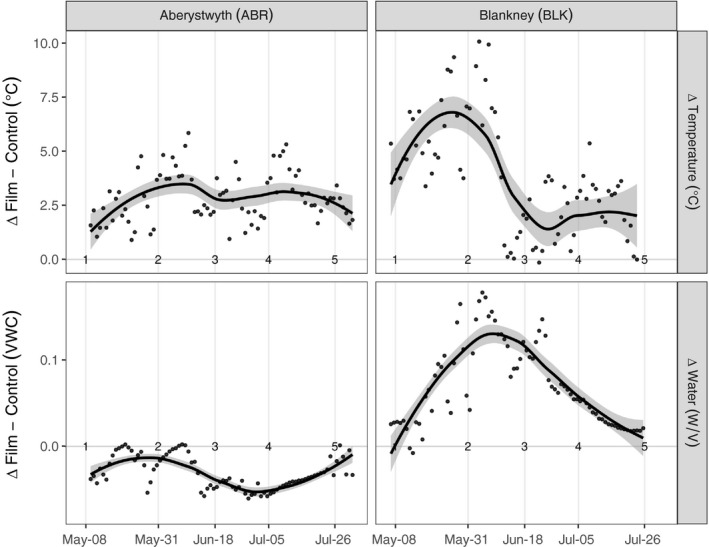
The effects of mulch film on soil surface (0–5 cm) temperature (top) and moisture (bottom), at two contrasting sites in the east and west of the United Kingdom. The difference (Δ) is calculated from averages of probe readings from plots covered with mulch film or control uncovered plots. Data are shown from the earliest sowing. Sowing dates are indicated by numbers (1–5) within the figures. A loess line has been applied to each set to show the trend in the data in black with a 95% confidence interval in grey (for illustrative purposes only)

Mulch film increased soil surface temperature during the first 2 months after it had been laid in both sites; after this, the mulch film began to degrade and the effects were greatly reduced. Soil surface temperature in control plots (without mulch film) was an average of 3.7°C warmer at BLK than ABR over the first five sowings (Figure 2). The greatest difference between sites was measured at sowing 4 when the temperature measured at BLK was 4.1°C higher under film than at ABR. The film produced a 2.7 ± 0.5°C daily mean increase in temperature in ABR and a 4.2 ± 0.6°C (±1 *SE*) daily mean in BLK. In BLK, the soil moisture content increased under mulch film compared to control plots and this continued until the film started to degrade. Soil surface moisture was more consistent under film because soil moisture losses were reduced compared with control plots. In ABR, the mulch film did not detectably increase the soil moisture content because sowing 1 in ABR (Figure 3) occurred during an unseasonably dry spring period (Figures S1 & S2).

### 3.2 Seedling emergence of field‐sown seed

**Figure 4:**
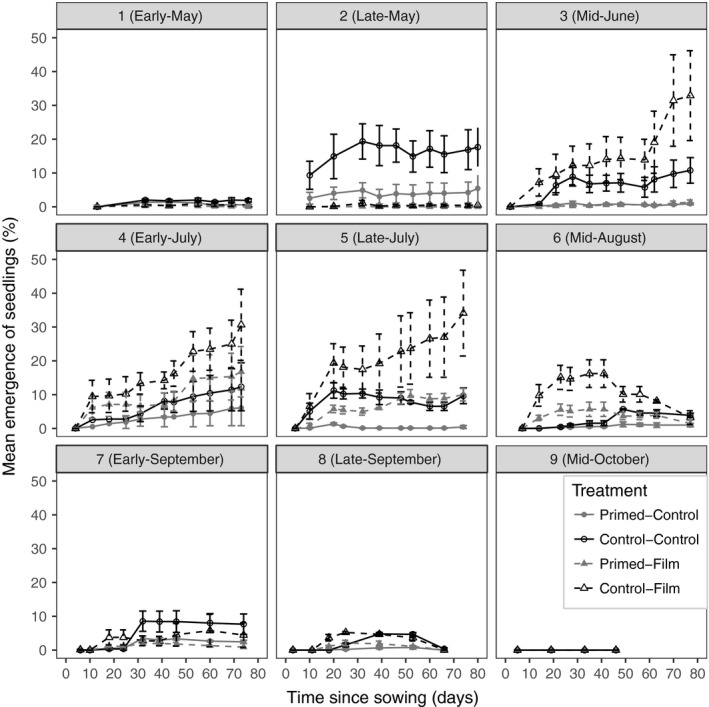
Seedling emergence counts for seed sown in Aberystwyth (ABR), over the first 80 days after each sowing at all nine sowings in 2013. Key shows treatment combinations. Errors are ±1 standard error

Seedling emergence counts at ABR for all nine sowing times in 2013 are shown in Figure 4. Of note is that emergence from the earliest sowing (1) and sowings 7 and 8 were below 10% in all treatments and sowing 9 did not germinate. The highest emergence percentage (35%) was recorded in sowing 5 (late‐July) for the unprimed, film‐covered treatment when both temperatures and soil moisture contents were highest. Emergence decreased in sowings after sowing 5, until sowing 9 when no germination was evident in 2013. Plots that had no emergence in the first year in ABR were assessed several times in the second year, and no seedlings were found. However in BLK, emergence in the second year occurred in the late‐sown plots (sowings 7 and 9) where no emerged seedlings were recorded in the first year. Primed seed that had not been covered by mulch film had the lowest levels of emergence across all sowing dates, other than in sowing 2 at ABR. The highest levels of emergence were recorded in control (nonprimed) seed covered by mulch film.

Comparing the likelihood ratios from generalised linear models for the emergence in the ABR site showed date of sowing and priming had a significant impact on the model (both *p* <* *.0001). This was not the case for mulch film where *p *=* *.14.

Mulch film had both a positive and negative effect on germination. In early sowings (1 and 2), mulch film‐covered plots had lower emergence, while in later successful sowings mulch film plots had higher emergence (Figure 4). The poor germination and emergence under film in sowings 1 and 2 in ABR were associated with low surface soil moisture content (Figure 3).

### 3.3 Seedling vigour from field‐sown seed

Tiller counts were averaged from three replicate plots per treatment per sowing time. At ABR, mean tiller counts ranged between 5 and 124 from film‐covered control seed, 0 and 59 from film‐covered primed seed, 1 and 146 from uncovered control seed, and, 1 and 40 from uncovered primed seed (Table 1). These values excluded sowings where there was no emergence in all three replicates.

**Table 1:** The mean tiller counts (tillers/m) of primed and control seedlings recorded in November of years 1 and 2 (2013–2014), sown both with and without mulch film, at both sites, in nine sowings. The tiller count data were not a normal distribution, and ±1 *SE* (standard error) is given (*n *=* *3)

Sowing no	Mulch film								Control							
	Primed				Control				Primed				Control			
	BLK		ABR		BLK		ABR		BLK		ABR		BLK		ABR	
	Avg.	*SE*	Avg.	*SE*	Avg.	*SE*	Avg.	*SE*	Avg.	*SE*	Avg.	*SE*	Avg.	*SE*	Avg.	*SE*
Year 1																
1 Early‐May	13	9.8	0	0.3	25	17.1	7	7.3	41	24.0	10	4.8	49	13.9	20	7.4
2 Late‐May	31	3.0	0	0.0	74	18.5	5	5.3	0	0.0	40	18.0	8	4.6	146	31.7
3 Mid‐June	63	31.6	4	2.2	73	2.3	124	53.0	11	9.1	6	1.8	5	4.2	47	10.4
4 Early‐July	5	2.5	59	29.8	5	1.7	97	35.5	0	0.0	20	15.9	0	0.0	33	16.3
5 Late‐July	7	4.3	38	8.4	25	6.7	93	27.9	0	0.0	1	0.6	4	2.6	20	4.7
6 Mid‐August	8	3.2	9	0.6	5	2.3	18	4.4	0	0.0	1	0.9	0	0.0	1	1.0
7 Early‐September	0	0.0	0	0.3	0	0.0	12	10.7	0	0.0	4	1.5	0	0.0	11	6.0
8 Late‐September	3	3.0	0	0.0	0	0.7	0	0.0	0	0.0	0	0.0	0	0.0	1	1.0
9 Mid‐October	0	0.0	0	0.0	0	0.0	0	0.0	0	0.0	0	0.0	0	0.0	0	0.0
Year 2																
1	51	35.8	0	0.0	102	60.7	2	1.7	99	53.5	23	11.7	135	65.4	53	25.6
2	77	7.5	0	0.0	147	37.7	22	22.3	0	0.0	65	13.9	32	17.9	235	31.6
3	86	43.4	5	4.7	206	42.0	208	54.0	37	24.7	0	0.0	16	15.7	92	13.0
4	17	17.3	99	49.7	4	4.3	147	68.6	0	0.0	39	34.6	0	0.0	70	50.7
5	0	0.0	42	24.5	37	18.5	121	37.7	0	0.0	0	0.0	0	0.0	14	2.2
6	0	0.0	4	4.0	0	0.0	12	4.6	0	0.0	0	0.0	0	0.0	0	0.0
7	3	3.3	2	2.0	0	0.0	4	1.2	0	0.0	0	0.0	0	0.0	1	0.7
8	0	0.0	0	0.0	0	0.0	0	0.0	0	0.0	0	0.0	0	0.0	0	0.0
9	4	2.1	0	0.0	0	0.0	0	0.0	0	0.0	0	0.0	2	2.0	0	0.0

Likelihood ratios were compared between negative binomial models of the number of tillers for both ABR and BLK. At ABR, priming and sowing time (both *p *<* *.0001) significantly affected tiller count. Control seed produced more tillers than primed seed by the end of the first year for all sowing dates at ABR. Mulch film did not have a significant effect on the model (*p *=* *.81).

At BLK, the model showed that mulch film and sowing date had a significant effect but priming did not (*p *<* *.0001, *p *<* *.0001, and *p *=* *.4). Mean tiller counts ranged from 1 and 74, 3 and 31, 4 and 49 and 11 and 41 for film‐covered control seed, film‐covered primed seed, uncovered control seed and uncovered primed seed, respectively. Mulch film had a negative effect on sowing 1 (early‐May), but when tested with a Kruskal–Wallis rank sum, this effect was not significant (*p *=* *.08).

Tiller count increased between years 1 and 2 in the majority of plots in which seeds germinated in year 1 (Table 1). In the later sowings, tiller number decreased in year 2; this may be attributed to overwinter seedling death due to lack of sufficient growth to develop rhizome needed to initiate growth in year 2. In ABR, sowing date and priming had a significant effect on second‐year tiller counts (*p *<* *.0001 and *p *<* *.0001) and the effect of film was not significant (*p *=* *.8). At BLK, a small number of seedlings emerged in year 2 in plots that showed no emergence from sowings 7 and 9 in year 1, suggesting that seed was able to remain viable and overwinter at this site.

At BLK, priming, sowing date and film had significant effects on tiller number in the second year when likelihood ratios were tested from negative binomial generalised linear models (*p *<* *.0001, *p *<* *.0001 and *p *<* *.01, respectively), with earlier sowings and film‐covered plots producing more tillers.

Tiller height in the year 1 sowings at both ABR and BLK was tested with a three‐way ANOVA, without interactions. At ABR, mulch film and sowing date had a significant effect on tiller height while priming did not (*p *<* *.01, *p *<* *.0001 and *p *=* *.68, respectively), the film treatment produced taller plants with sowing around June producing the tallest plants. At BLK, only sowing date, which produced the tallest plants from seed sown in late‐May, had a significant effect on tiller height which was not significantly affected by either mulch film or priming (*p *<* *.0001, *p *=* *.27 and *p *=* *.34, respectively).

A Tukey's HSD placed sowings 1–5 at ABR in the high means group, sowings 5 and 6 in the intermediate group and sowings 6–8 in the low group. Seedlings from seed sown during or before July at ABR (high group) had mean tiller heights of >60 mm, while sowings after July had heights of <30 mm. Tiller heights in film‐covered plots were on average 23 mm ± 12 mm (*SE*) greater than uncovered plots. The same analysis for BLK showed an upper grouping of sowings 1–3 (May/June) with heights >160 mm and a lower grouping of sowings 4–6 (July/August) with heights ≤60 mm. Seedlings from seed sown after September at BLK (sowings 7–9) did not have a measurable tiller height.

Tiller diameter was significantly affected by sowing date in ABR and BLK when tested with a three‐way ANOVA (*p *<* *.0001 and *p *<* *.0001) (Table 2). Mulch film and priming had no significant effect (*p *=* *.83 and *p *=* *.15, respectively, at ABR; *p *=* *.15 and *p *=* *.95 at BLK). Tukey's HSD analysis identified two groupings at ABR; sowings 1 (early‐May) through to 5 (late‐July) had mean diameters ranging between 3.2 and 4.3 mm, while the lower grouping of sowings 4 (early‐July) to 8 (late‐September) had mean diameters of 1–3.2 mm. At BLK, sowings were placed into three groups; 1 and 2 (May) had the thickest stems (mean diameter >5.5 mm), sowings 1 and 3 were placed in the intermediate group, and 4–6 (July to August) produced the thinnest stems (mean diameter <2 mm). Mean number of leaves per tiller followed the same pattern as tiller diameter at both sites. A three‐way ANOVA produced the following results: *p *<* *.0001, *p *=* *.16 and *p *=* *.36 at ABR and *p *<* *.0001, *p *=* *.32 and *p *=* *.99 at BLK for sowing date, mulch film and priming, respectively.

**Table 2:** The mean tiller diameters (mm) of primed and control seedlings recorded in November of years 1 and 2 (2013–2014), sown both with and without mulch film, at both sites, in nine sowings. The tiller diameter data were normal distributed, and ±1 *SE* (standard error) is given (*n *=* *3)

Sowing no	Mulch film								Control							
	Primed				Control				Primed				Control			
	BLK		ABR		BLK		ABR		BLK		ABR		BLK		ABR	
	Avg.	*SE*	Avg.	*SE*	Avg.	*SE*	Avg.	*SE*	Avg.	*SE*	Avg.	*SE*	Avg.	*SE*	Avg.	*SE*
Year 1																
1 Early‐May	5.4	1.1	0	0	7.2	0.5	5.2	0	4.7	1.3	4.6	1.1	5.3	0.3	3.5	0.8
2 Late‐May	7.4	1	0	0	6.1	0.4	5.5	0	0	0	4.2	0.8	5.3	0.6	3.8	0.3
3 Mid‐June	5	0.2	4	0.8	5.2	0.6	3.8	0	4.3	0.6	4.8	0.4	3.7	1.7	4.3	0.2
4 Early‐July	2.2	0.5	3.5	1.2	1.5	0.3	2.9	0.8	0	0	2.9	0.9	0	0	3.1	0.6
5 Late‐July	1.7	0.2	3.9	0	2.1	0.2	3.4	0.3	0	0	2	0.8	1.8	0.4	3.1	0.7
6 Mid‐August	1.2	0.2	2.3	0.4	1.1	0.1	1.9	0.2	0	0	1.2	0.6	0	0	1	0
7 Early‐September	0	0	3	0	0	0	1.1	0	0	0	2.4	1.3	0	0	1	0
8 Late‐September	0	0	0	0	0	0	0	0	0	0	0	0	0	0	1	0
9 Mid‐October	0	0	0	0	0	0	0	0	0	0	0	0	0	0	0	0
Year 2																
1	8.5	0.1	0	0	9	0.5	9	0	7.4	0.6	12.8	1.4	8.5	0.9	9.6	0.5
2	7.7	0	0	0	8.5	0.6	9	0	0	0	8.5	0.2	7.4	0.7	7.5	0.3
3	6.4	0.3	11	0	7.3	0.4	9.3	1.3	6.9	0.1	0	0	8.4	0	9.8	0.8
4	6.6	0	9.3	0.3	10	0	7.5	1.2	0	0	8.6	0.5	0	0	9.4	0.4
5	0	0	8.1	1.2	5.2	0.6	8.2	0.6	0	0	0	0	0	0	8.3	1.2
6	0	0	6.5	0	0	0	7.9	0.6	0	0	0	0	0	0	0	0
7	5	0	3.5	0	0	0	4	0.8	0	0	0	0	0	0	4	0
8	0	0	0	0	0	0	0	0	0	0	0	0	0	0	0	0
9	5	0.8	0	0	0	0	0	0	0	0	0	0	6	0	0	0

As expected, tiller height, diameter and leaf count were all greater in year 2 than in year 1 at both sites (Table 3). Sowing date had a significant effect when tested with a three‐way ANOVA, indicating a significant effect on tiller height, tiller diameter and leaf count *p *<* *.001, *p *<* *.0001 and *p *<* *.001, respectively) at ABR. Priming had no significant effect on tiller height, tiller diameter or leaf number (*p *=* *.8, *p *=* *.136 and *p *=* *.94, respectively). Mulch film only significantly affected tiller diameter (*p *<* *.05) but not tiller height or leaf number (*p *=* *0.51 and *p *=* *.303, respectively). At BLK, tiller height, tiller diameter and leaf count were all significantly affected by sowing date (*p *<* *.0001, *p *<* *.001 and *p *<* *.001, respectively). Mulch film had no significant effects (*p *=* *.854, *p *=* *.405 and *p *=* *.783, respectively), and priming only significantly affected tiller diameter (*p *<* *.05) but not tiller height or leaf number (*p *=* *.916 and *p *=* *.622).

**Table 3:** The mean tiller height (cm) of primed and control seedlings recorded in November of years 1 and 2 (2013–2014), sown both with and without mulch film, at both sites, in nine sowings. The tiller height data were normal distributed, and ±1 *SE* (standard error) is given (*n* = 3)

Sowing no	Mulch film								Control							
	Primed				Control				Primed				Control			
	BLK		ABR		BLK		ABR		BLK		ABR		BLK		ABR	
	Avg.	*SE*	Avg.	*SE*	Avg.	*SE*	Avg.	*SE*	Avg.	*SE*	Avg.	*SE*	Avg.	*SE*	Avg.	*SE*
Year 1																
1 Early‐May	13.2	9.2	0	0	21.4	3.6	13.2	0	18	7.9	8.5	1.3	19.5	5.8	6.5	1.3
2 Late‐May	27.7	1.9	0	0	25.6	5.5	10	0	0	0	11.9	4	15.5	2	10.5	1.2
3 Mid‐June	23	1.5	8.1	0.5	22.4	1.5	13.1	2.9	12.3	2.7	7.5	1.9	6.5	4.5	9.1	2
4 Early‐July	6.8	0.7	10.4	4.6	3.8	0.8	9.8	3.3	0	0	5.8	2.9	0	0	6	3
5 Late‐July	4.5	0.6	7.3	0.5	4.2	0.2	10.2	1.4	0	0	2.5	0.4	10.5	5.5	3.7	1.3
6 Mid‐August	1.7	0.1	3.8	0.3	1.8	0.1	3.5	0.1	0	0	0.6	0.1	0	0	1.2	0
7 Early‐September	0	0	3	0	0	0	2.3	0.2	0	0	0.6	0.1	0	0	0.6	0.2
8 Late‐September	0	0	0	0	0	0	0	0	0	0	0	0	0	0	0.3	0
9 Mid‐October	0	0	0	0	0	0	0	0	0	0	0	0	0	0	0	0
Year 2																
1	87	21.2	0	0	99.7	2.1	9	0	97.8	6.2	36.6	4.4	90.8	10.5	22.7	5.9
2	83.3	12.3	0	0	79	7.2	38.7	0	0	0	39.3	3.8	46.5	3.1	51.4	5.7
3	89.4	8.6	19.5	0	84.5	16.7	47.9	4.6	50.6	10.4	0	0	62.5	0	30.9	10.1
4	42.2	0	46.6	2	34	0	37.5	13	0	0	23.2	9.6	0	0	25.5	13
5	0	0	19.8	1.7	19.8	0.3	22.9	2.1	0	0	0	0	0	0	15.9	6.6
6	0	0	16.5	0	0	0	17.5	0.7	0	0	0	0	0	0	0	0
7	10	0	3.5	0	0	0	12.8	4.3	0	0	0	0	0	0	4	0
8	0	0	0	0	0	0	0	0	0	0	0	0	0	0	0	0
9	18.5	3.7	0	0	0	0	0	0	0	0	0	0	16.5	0	0	0

### 3.4 Thermal germination response in the lab

**Figure 5:**
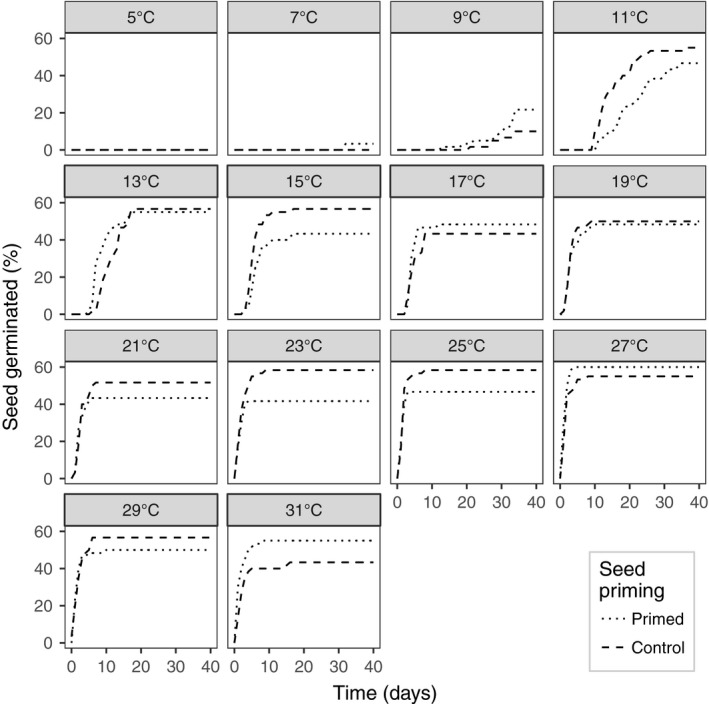
The cumulative proportion of *Miscanthus sinensis* seeds from primed and control (unprimed) treatments that germinated on a thermal gradient plate with 14 static temperatures over a range of 5°C (coolest) to 31°C (warmest)

Seed viability was consistent across the temperature treatments (Figure 5). The thermal time to 50% viable seed germination was not different between primed and unprimed seed; however, there was a small increase in germination in primed seed at 7 and 9°C (Figure 4). The base temperature (*T*_b_) for germination was estimated (Gardarin, Guillemin, Munier‐Jolain, & Colbach, 2010; Gummerson, 1986; Trudgill, Squire, & Tompson, 2000) using a linear regression model as described by Clifton‐Brown et al. (2011). The *T*_b_ for primed seed was 1.2°C higher than for unprimed seed (6.98 and 5.74°C, respectively). However, the upper and lower 95% confidence intervals for primed and unprimed were 8.02–5.34°C and 7.35–3.32°C, respectively.

## 4 DISCUSSION

Christian et al. (2005) found that *M. sinensis* seedlings could establish and survive the first winter in the United Kingdom. *M. sinensis* has been shown to survive well in cooler climates when compared with *M. sacchariflorus* and several *M*×*g* types (Clifton‐Brown & Lewandowski, 2000; Clifton‐Brown et al., 2001). *M. sinensis* seed is larger (~1 mg) than *M. sacchariflorus* seed (~0.6 mg) and is easier to produce in large quantities. For these reasons, we decided to evaluate *M. sinensis* seed from a recently bred synthetic population based on five parents obtained from China and Japan in 2008 which were selected in the continental climate of Indiana, USA, to develop the “know‐how” to perform direct field sowing in the United Kingdom with a range of sowing times, mulch films and priming. Because the synthetic population used was produced from five geographically diverse parents, this seed is representative of *M. sinensis*.

### 4.1 Sowing time

We hypothesised that plants from earlier sowings would outperform plants from later sowings, in the first and subsequent growing seasons. As expected, we found sowing time had a significant effect on germination, seedling growth characteristics and stand establishment success. It was counterintuitive that some of the earliest sowings would germinate so poorly when previously Christian et al. (2005) achieved successful germination of *M. sinensis* in April. However, the seedlings that did germinate were consistently larger at the end of the first growing season than were those from later sowings. Plants from earlier sowings were larger than those from later sowings in the second year of growth. This suggests that the longer growing season available to earlier sowings improved overwintering and regrowth. Our results appear consistent with Robson et al. (2013) in which it was shown that canopy duration was positively correlated with above‐ground yield across a large and diverse population of *Miscanthus*. However, Zub, Rambaud, Béthencourt, and Brancourt‐Hulmel (2012) showed later emergence and rapid growth maximised development in *Miscanthus*. Both studies used rhizome not seed propagation, but the complex interactions of early season environmental factors are illustrated by the different conclusions evident from these studies. This is certainly reflected in our study in that the positive impact of early growth and subsequent longer season is seen in the increased biomass per plant, but the negative impact is seen in the poor initiation and maintenance of germination and growth.

*Miscanthus* is a rhizomatous grass and plants from earlier sowings have a longer growing season, which is needed to grow the rhizomes, which store carbohydrate. Rhizomes are needed for overwintering, and stored carbohydrate is remobilised to drive spring regrowth in the following year. Biomass and canopy senescence were not found to be correlated with overwinter survival in *Miscanthus* (Clifton‐Brown & Lewandowski, 2000); however, stay‐green *Miscanthus* survived poorly in higher latitudes in Europe (Clifton‐Brown et al., 2001) and first‐year *M *× *g* overwintered poorly in a study in the United States and maintained active leaves after late‐season frosts, suggesting a lack of senescence may be responsible (Boersma, Dohleman, Miguez, & Heaton, 2015). We anticipate the production of rhizome and the timing of senescence are critical factors in overwinter survival and that this is strongly linked to canopy duration in particular during the first year of establishment. In our field experiment, we did not measure rhizome growth from the different sowing times because the seeds were sown too densely to separate plants reliably. Our experiment could not explore the longer term planting density effects on yield, because the plots were small single rows. These are both important aspects that need to be included in further seed establishment studies.

The peak of germination in sowings 3 and 5 (mid‐May to late June) and decline after sowing 5 demonstrate germination in *Miscanthus* seed is clearly impacted by both seedbed temperature and moisture content, two factors that are dynamic throughout the growing season. Soil temperatures will limit germination and growth from early sowings, while soil moisture may limit growth from later sowings. Meteorological data collected over both years of the trial showed that at both sites low rainfall in June and July resulted in drier soil surfaces in uncovered plots. The effect of this is likely to be compounded by high average temperatures at this time of year. Christian et al. (2005) found that high temperatures and low rainfall after seedling emergence lead to seedling dessication and death. The data from this single‐year, two‐site experiment suggest an optimum sowing time of late‐May to early‐June for *Miscanthus* in the United Kingdom. Sowing late‐May to early‐June would utilise the majority of the growing period allowing good rhizome development while ensuring that soil temperatures were suitable to ensure good germination. Sowing later to ensure higher seedbed temperatures risks poorer germination due to low soil moisture. To ensure soil moisture is as far as possible retained sowing should be soon after cultivation, possibly by following the cultivator with the seed drill and covering immediately with mulch film.

### 4.2 Mulch film

Mulch film has the potential to provide a number of advantages to field‐sown crops including protection from pests such as rabbits by allowing seedlings to develop more biomass before exposure to herbivory (Bégin, Dubé, & Calandriello, 2001; Lamont, 2005). However, this study focused on quantifying the impact of mulch film on temperature and soil moisture which are key factors for germination and early plant development. Mulch film increased yield in maize crops and allowed maize to be sown at increased plant densities (Liu et al., 2014; Qin, Hu, & Oenema, 2015). In a previous study, the impact of temperature on germination was a major limitation on the modelled geographical area available to direct‐sown *Miscanthus* (Clifton‐Brown et al., 2011). The thermal gradient experiment showed that optimum temperatures for germination for this *M. sinensis* seed batch were >11°C, similar to earlier published values (Clifton‐Brown et al., 2011; Hsu, 1985). In the field trials, mulch film provided an average temperature increase of 3°C. We hypothesised that mulch film would increase germination in all sowings, but particularly in early spring sowings. Unexpectedly, we did not observe a beneficial impact on the germination rates of early sowings. Soil surface temperature measurements showed the mulch film prevented the temperature from declining below 0°C while the temperature in the control plots was 0°C or less. This would provide effective frost protection for any “just‐germinated” seedlings. However, the temperature enhancement under the film in the earliest sowings was insufficient to positively impact germination rates.

Mulch film can increase soil moisture by preventing evaporation and elevate deep water to more accessible depths (Wang, Li, Song, & Li, 2003; Zhou et al., 2009). As expected, film‐covered plots had higher soil surface moisture contents, and reduced fluctuations in soil moisture were recorded at BLK. However, film laid in early spring on dry soil in ABR resulted in lower soil moisture content than uncovered plots (Figure 3). The capture of evaporation or runoff water did not raise the soil surface moisture content as detected by the soil moisture reflectometers near the middle of the plot. The seed was sown in the middle of the plot which was presumably the driest area of the plot. Such spatial problems in available water may be addressed using a system of ridges (Li & Gong, 2002; Zhou et al., 2012) and sowing at the edge of ridges which may aid rain flow to the roots. From late‐May onwards, germination was not limited by temperature. Here, the film, if laid shortly after rainfall, had a positive impact on germination. However, it is likely that soil and air temperature under film could be harmful at later sowing dates. Results from trials on tomatoes showed that high root zone temperatures enhanced by mulch film reduced yield (Díaz‐Pérez & Batal, 2002).

While sowing under film does not reduce the risk of poor germination, it does accelerate the growth of germinated seedlings. In the second year, biomass was significantly increased in plots that had been covered by film, indicating that growth gains in year 1 were continued into the subsequent season. If germination rates could be improved by protocol developments such as sowing near the film edges to get better access to water to achieve more consistent germination from seed, the additional cost of mulch film ~£300 per hectare (Hastings et al., 2017), when used in suitable environmental conditions, is likely to be financially beneficial.

### 4.3 Priming

In our thermal gradient experiments, priming seed had little effect on *T*_b_; however, field emergence and tiller counts in both years were significantly reduced by priming. This was not translated into a significant negative effect on establishment measurements in the field. Despite the positive effect of priming in other crops, all but the 9°C thermal gradient plate treatment showed priming to have a negative or neutral effect on germination in the *M. sinensis* hybrid used here. Incorrect priming of seed can easily reduce seed germination (Arif, Jan, Marwat, & Khan, 2008), while the dried primed *Miscanthus* seed did germinate the seed lacked the vigour often associated with priming and while this seed was primed by an experienced seed technology company (Elsoms Seeds Ltd.) this was the first *Miscanthus* seed primed and therefore further optimisation may be possible.

### 4.4 Location

First‐ and second‐year plants at BLK were significantly taller with more shoots per plant than those at ABR. However, fewer plots established in the first year at BLK than at ABR (47 of 108 plots (or 43.5%) and 68 of 108 plots (or 63%), respectively). Higher‐than‐average temperature in BLK aided growth compared to ABR, however rainfall limited germination and establishment. Soil texture at BLK is lighter than at ABR, and while light‐textured soils may improve early plant development rates (Passioura, 2002), heavier soils have been associated with improved yield over lighter soils in *C*_4_ grasses (Potter, Bingham, Baker, & Long, 1995) and in *M *× *g* specifically (Maughan et al., 2012).

### 4.5 Potential of direct sowing for *Miscanthus*

To establish perennial *Miscanthus* directly from seed involves overcoming often conflicting environmental limitations to germination and seedling growth involving temperature, availability of water and growing season duration. The differing environmental conditions in the sites in the west and east of the United Kingdom revealed many complex interactions between agronomy and local conditions.

Priming had no significant positive effects on the germination, field emergence or establishment of *Miscanthus*. The lack of herbicides was a limitation of this study; this meant film also accelerated weed growth. Sowings earlier than May were not studied, but germination and morphological parameters associated with yield increased from low levels for seed sown in May to a peak during July, after which germination rates and growth declined.

Mulch films did not increase overall germination rates but accelerated early plant growth, reducing the risk of overwinter plant failure in the first year which is known to speed up the time to reach mature crop yield levels. Low temperatures in the ABR site limited growth, and the elevated temperatures under mulch film were insufficient to exceed the basal thermal requirements for germination of the seed to improve the germination rates in early sowings. Soil moisture, particularly when interacting with the mulch film and sowing day, had a larger effect on emergence of seed and overall success of sowings. Water deficits severely limited germination in the field, both at ABR, where early sowings were onto dry soil, and at BLK where the drier climate resulted in fewer surviving plots.

Approximately 20% of field‐sown seed successfully germinated, established and overwintered in late‐May sowings. This showed the potential for establishing *Miscanthus* by direct‐sown seed in the United Kingdom, but at this level, the rate of oversowing required to establish complete field canopies would probably be uneconomic. Improved protocols for direct sowing are needed to increase the efficiency of seed usage before this can become a commercial prospect.

This sowing experiment was only performed on one synthetic *M. sinensis* cross; therefore, the results may not reflect the range of responses in other *Miscanthus* seed lots in a UK climate in regard to sowing time, priming or film. Further experiments are needed to refine the agronomic techniques for commercial establishment of seed‐based direct‐sown crops of *Miscanthus* and may require a combination of genetic improvement and application of film and physical seed treatments.
