# Supplementary material for: Assessing seed priming, sowing date, and mulch film to improve the germination and survival of direct‐sown Miscanthus sinensis in the United Kingdom
Source: Glob Change Biol Bioenergy. 2018 Jun 7;10(9):612–27. doi: 10.1111/gcbb.12518 (PMC6473505; doi:10.1111/gcbb.12518)
Supplement: Supplementary file 1 [file GCBB-10-612-s001.pdf]

SUPPLEMENTARY FIGURE S1

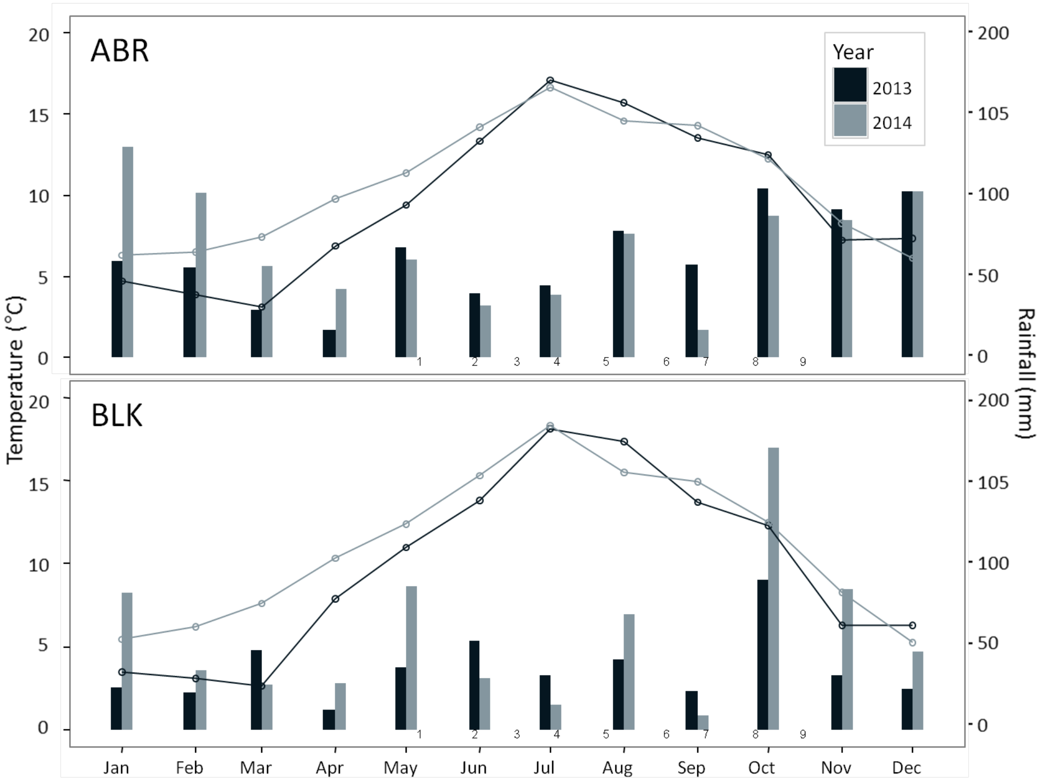

Monthly rainfall totals (mm) in 2013 and 2014 and mean monthly air temp (°C) for trial sites at Aberystwyth (ABR) and Blankney (BLK). Bars represent rainfall, lines represent temperature. The left hand y-axis shows rainfall (mm) and right hand y-axis shows air temperature (°C).

SUPPLEMENTARY FIGURE S2

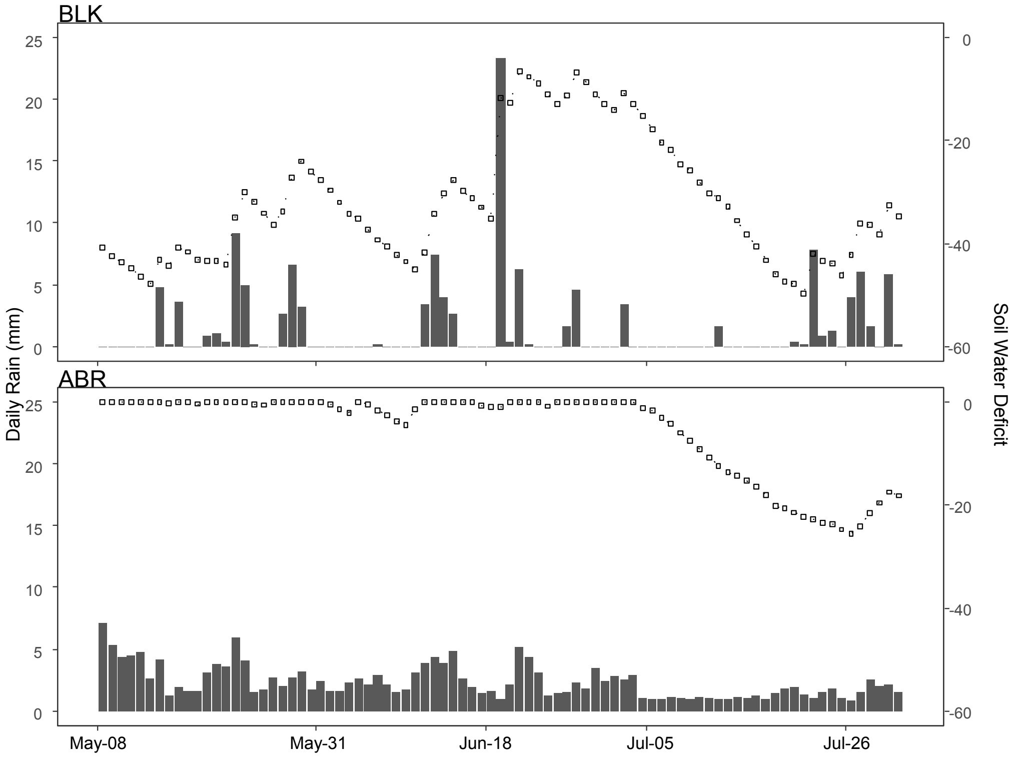

Mean daily precipitation [bars], and calculated soil water deficit [□] (calculated from January 1st 2013) are for both field sites.
